# Basal Cell Carcinoma of the Head and Neck Region: An Analysis of 171 Cases

**DOI:** 10.1155/2012/943472

**Published:** 2012-12-19

**Authors:** Omer Sefvan Janjua, Sana Mehmood Qureshi

**Affiliations:** ^1^Department of Oral and Maxillofacial Surgery, Dental Section, Punjab Medical College, Faisalabad 38000, Pakistan; ^2^Department of Oral and Maxillofacial Pathology, Dental Section, Punjab Medical College, Faisalabad 38000, Pakistan

## Abstract

*Objective*. To analyze the pattern of presentation of basal cell carcinoma (BCC) and margin status for excised specimens in the head and neck region. *Study Design*. Retrospective cross-sectional. *Duration of Study.* January 2009 to December 2011. *Methodology*. The database of the pathology department was searched to identify records of all malignant skin tumors that underwent standard excision with margins. Out of these records, tumors with a diagnosis of BCC in the head and neck region were retrieved and separated. Age, gender, anatomic location, pattern of tumor, and margin status were noted. *Results*. A total of 171 cases of BCC from various sites of head and neck were retrieved. Male to female ratio was 1.4 : 1. The age ranged from 22 to 90 years. Seventy-six cases presented on right side, 79 on left, and 16 were in the midline. Most common anatomical site was the nose followed by the cheek. Nodular lesions were the most common (46.2%) followed by pigmented variety (18.7%). Margins were clear in 77 (45.1%) cases, involved in 86 (50.2%) cases, and close in 8 (4.7%) cases. *Conclusion*. Nose was the most common site followed by the cheek. Nodular and pigmented varieties were the most frequent and margins were involved in more than fifty percent of the cases.

## 1. Introduction

Jacob Arthur in Dublin in 1827 first coined the term “rodent ulcer” to describe what we now know as a basal cell carcinoma (BCC) [1]. It takes its name from the resemblance of the epithelial tumor cells to normal basal cells of the skin. Basal cell carcinoma is a locally invasive, slow growing malignancy that arises from the basal layer of the epidermis and its appendages and is considered to be most common skin malignancy in adults of fair-complexion [2, 3]. Recent studies have shown that two thirds of the tumors are located in the head and neck region as these are the most sun exposed regions of the body [4]. The incidence of BCC has increased substantially in the last decade or so and it shows geographical variations [5].

Prolonged exposure to ultraviolet light is the chief cause of development of BCC [6]. Other predisposing factors include exposure to radiotherapy, arsenic, albinism, burns, scars, immunosuppression, Bazex, and Gorlin syndromes [7–10].

Clinically, BCC in *the* head and neck usually presents as a slow growing well defined papule *or* nodule with telangiectasias located above the line connecting the angle of the mouth and ear lobe. It is a locally destructive lesion and can cause serious disfigurement but metastasizes rarely. According to clinical appearance and aggressiveness there are several variants including nodular (with or without ulceration), pigmented, sclerosing, superficial, and baso-squamous [11, 12]. 

Histologically, BCC exhibits uniform, extremely dark basaloid cells with little cytoplasm and oval nuclei. The cells are attached to the epidermis and reach downward into the papillary dermis. There are distinct islands and cords of cells with palisading or “picket fence” arrangement of the peripheral cells that mimics the basal layer of the skin. Some lesions produce keratin with pearl formation and in others benign melanocytes give the lesion a black color [7, 13].

Surgical resection with an adequate margin of normal tissue is the accepted standard of practice. Reported recurrence rates are around 10% when a BCC is completely excised as compared to recurrence rates of over 30% when a BCC is incompletely excised. Clinicians usually employ the technique of Moh's micrographic surgery to achieve margin clearance in BCC [14, 15].

The rationale for the study was to evaluate the pattern and presentation of basal cell carcinomas of head and neck areas in northern Pakistan along with the evaluation of frequency of specimens where margin clearance was obtained. 

## 2. Methodology

Data of all the malignant skin tumors which presented to the Pathology department from January 2009 through December 2011 were analyzed retrospectively. Out of these records, tumors with a diagnosis of basal cell carcinoma were retrieved. These cases were narrowed to BCC that occurred in the head and neck region. Hematoxylin and eosin stained slides were examined under the microscope and histopathological pattern was reconfirmed on these slides. The age, gender of the patient, and anatomic location and pattern of tumor were noted as described in the records. The samples with incomplete record or with necrosed, scanty, and autolysed tissues were excluded from the study as it was difficult to identify the clinical parameters and histopathological pattern in these samples. 

The tumor was labeled as nodular BCC if it comprised of well demarcated islands and strands which arose from basal cells of the epithelium and contained uniform ovoid, dark staining basaloid cells with moderate sized nuclei and relatively little cytoplasm. If the sample consisted of tumor islands containing dendritic melanocytes and melanophages, it was labeled as pigmented BCC. When strands of basaloid tumor cells were present with a densely collagenous background, it was classified as sclerosing variety. If lobules of tumor cells that dropped from epidermis in a multifocal pattern were present, the lesion was termed as superficial BCC. When BCC was admixed with squamous cell carcinoma of the skin we labeled it as baso-squamous variety. 

 In order to assess the margins status of the excised specimens, the formalin fixed tissue specimen was examined grossly and then oriented for histopathology examination. The margins were painted with India ink and examined under the microscope. Margins were considered clear when no tumor was present within 3–5 mm of the resection margin, close when tumor existed within 1 mm and involved when the tumor encroached on the resection margins and the distance was either less than 1 mm or there was a cut through *the* tumor.

## 3. Statistical Analysis

All data was recorded on specially designed forms developed for the data collection and analyzed using SPSS version 17.0. Mean and standard deviation was calculated for age of presentation and size of the lesion. Frequency and percentages were calculated for gender, pattern of presentation, and margin status. 

## 4. Results

A total of 171 cases of BCC from various sites of head and neck were retrieved. Out of these, 100 (58.5%) patients were male and 71 (41.5%) were female with an overall male to female ratio of 1.4 : 1. The age ranged from 22–90 years (mean 61.3 ± 13.07 years). Seventy six of the cases were located on the right side of face, 79 on left side while 16 were in the midline.

The most common anatomical site was *the* nose where tumor occurred in 53 (31.5%) cases followed by *the *cheek which was involved in 46 (26.9%) cases. Regarding pattern of presentation, nodular lesions were the most common, seen in 79 (46.2%) cases followed by pigmented (32 cases, 18.7%) and superficial (31 cases, 18.1%) varieties, respectively. Complete description of anatomical sites and patterns is presented in Table 1. The size of the tumor specimens was also analyzed from the records of the gross specimen and ranged from 0.2 cm to 9.0 cm (mean 2.2 ± 1.4 cm). 

**Figure 1:**
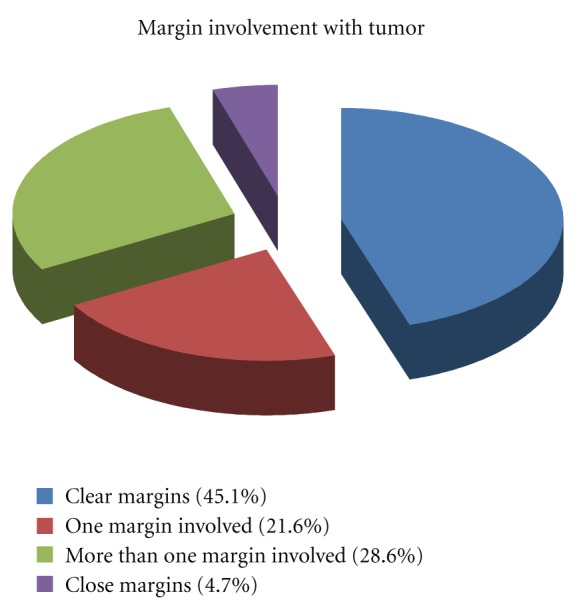
Showing margin clearance for BCC.

We also assessed margin clearance in the excised specimens and it was seen that in 77 (45.1%) cases margins were clear, in 37 (21.6%) cases one of the margin was involved, in 49 (28.6%) cases more than one margin was found involved while in 8 (4.7%) cases margins were found to be close (Figure 1). Ear, nose and eyelids were the sites that showed maximum involvement of margins in the resected specimens respectively. 

Chi square test was used to determine the effect of site on margin clearance but the results obtained were statistically insignificant. (*P* value >0.05).

## 5. Discussion

Basal cell carcinoma (BCC) is considered to be the most common skin malignancy and its incidence is on the rise worldwide because of increased exposure to UV light and ozone depletion in various parts of the world due to environmental and industrial pollution [16]. Exposure to UV light results in DNA damage that leads to development of skin cancer [17]. Similarly patients with immunosuppression are more prone to develop nonmelanoma skin cancers. BCC has been linked with various syndromes also like Bazex, Rombo, Nevoid Basal Cell Carcinoma Syndrome, Rasmussen, and Darier's disease because of mutations in Sonic hedgehog pathway [3, 18]. 

In our study, BCC was slightly more common in male patients (M : F = 1.4 : 1). This could be because of the fact that males are more exposed to sun light because of their outdoor occupations. These results are similar to what is reported by Aandani and Ganatra [4] and Cigna et al. [19] while on the other hand Chow et al. [5] reports BCC to be more prevalent in females. BCC was most prevalent in 6th and 7th decade in our study group and mean age of presentation was 61 years. Similar results were reported by Cigna et al. [19] while Aandani and Ganatra [4] reports BCC to be more prevalent in 5th decade in their study.

When we analyzed the anatomical site for occurrence of BCC, we found that *the* nose was the most common location followed by cheek. These results are in harmony with those reported by Chow et al. [5] and Aandani and Ganatra [4] who also report *the* nose and *the* cheek to be the most commonly involved site for BCC.

 Another factor that we assessed in our study was the histopathological pattern of the tumor and in this regard, we found that nodular variety was the most frequent in our study sample followed by the pigmented variety. Aandani and Ganatra [4] and Cigna et al. [19] also report ulcerative and nodular variety to be the most common type of pattern. 

 From our records we extracted the information about the size the lesions and it was seen that the mean size at the time of excision was approximately 2 cm ranging from 0.2 cm to 9 cm. Cigna et al. [19] from Italy, reports a slightly smaller size of lesion at the time of presentation, around 12 mm. Late presentations for malignancy are a common finding in patients of third world countries [20]. 

One of the fundamental requirements for treating BCCs or for that matter any malignancy is attainment of clear surgical margins after complete excision. So we assessed our samples for margin clearance and it was found out that we could achieve margin clearance in only 45 percent of the cases while in around 50 percent cases either one of more than one margin was involved by the tumor. These results are extremely high as compared to what is reported in the literature. Studies report frequency of incomplete excision ranging from 3–20% [1, 14, 15, 21]. A four millimeter excision margin is currently recommended for small, well demarcated BCC's as this gives a complete excision rate of approximately 95% [22]. Ear, nose, and eyelids were the sites where involvement of margins was seen most of the time in our cases. Possible reasons for that could be unavailability of Mohs micrographic surgery in most of the plastic and maxillofacial units, and in these areas anatomical landmarks are pressing and surgeons tend to go less aggressive while excising tumors. Another possibility could be larger sized tumors at the time of presentation. For cases with involved margins either reexcision or radiotherapy was advised. Re excision was done mostly in cases where site was closed primarily and in cases where some flap was used, the patients were referred for radiotherapy. 

## 6. Conclusion

BCC is quite common in our society. *The* nose was the most common site followed by *the* cheek. Nodular and pigmented varieties were the most frequent. Margin clearance could not be achieved in more than fifty percent of the cases. All effort should be directed towards achievement of clear margins in order to decrease the chances of recurrence and improve survival. Attempts should be made to utilize Moh's micrographic surgery whenever available or one can attempt staged excision.

## Figures and Tables

**Table 1:** Showing association of pattern and site along with their frequencies and percentages.

Site	Pattern					Total
	Nodular	Pigmented	Sclerosing	Superficial	Baso-squamous
Angle of mouth	1	0	0	0	0	**1 (0.6%)**
Cheek	19	8	7	6	6	**46 (26.9%)**
Chin	0	0	0	2	0	**2 (1.2%)**
Ear	0	2	2	2	0	**6 (3.5%)**
Forehead	6	2	0	3	2	**13 (7.6%)**
Lower eyelid	9	0	0	7	0	**16 (9.4%)**
Lower lip	2	0	0	0	1	**3 (1.8%)**
Neck	3	0	0	1	0	**4 (2.3%)**
Nose	28	9	2	6	8	**53 (31%)**
Scalp	2	3	0	1	0	**6 (3.5%)**
Supra orbital	0	2	0	0	0	**2 (1.2%)**
Temporal	2	3	0	2	0	**7 (4.1%)**
Upper eyelid	5	2	0	1	0	**8 (4.7%)**
Upper lip	2	1	0	0	1	**4 (2.3%)**

Total	**79 (46.2%)**	**32 (18.7%)**	**11 (6.4%)**	**31 (18.1%)**	**18 (10.5%)**	**171 (100%)**
